# Species delimitation of *Margattea* cockroaches from China, with seven new species (Blattodea, Ectobiidae, Pseudophyllodromiinae)

**DOI:** 10.3897/zookeys.1036.63232

**Published:** 2021-05-10

**Authors:** Jia-Jun He, Du-Ting Jin, Yi-Shu Wang, Yan-Li Che, Zong-Qing Wang

**Affiliations:** 1 College of Plant Protection, Southwest University, Beibei, Chongqing 400715, China Southwest University Chongqing China

**Keywords:** ABGD, bPTP, cockroaches, COI, GMYC, intraspecific difference, morphology

## Abstract

Nearly 450 *Margattea* specimens were collected from 27 locations in China and their morphology was examined. Then 68 *Margattea COI* sequences were obtained and used to carry out phylogenetic analyses as well as species delimitation analyses using General Mixed Yule Coalescent (GMYC), Automatic Barcode Gap Discovery (ABGD), and Poisson Tree Processes (bPTP). GMYC analysis resulted in 21 molecular operational taxonomic units (MOTUs) (confidence interval: 20–22), which was completely consistent with the result of the bPTP. There were 15 MOTUs using the ABGD method. The number of MOTUs was slightly different from the assigned morphospecies (16). As to the incongruence between molecular and morphological results, we checked the specimens again and made sure that most morphological differences were determined to be intraspecific differences (except the difference between *M. angusta* and *M. mckittrickae*), although a large genetic distance existed. Finally, 16 *Margattea* species from China were defined in this study, of which, seven new species are established, i.e. *Margattea deltodonta* J-J He & Z-Q Wang, **sp. nov.**, *Margattea cuspidata* J-J He & Z-Q Wang, **sp. nov.**, *Margattea caudata* J-J He & Z-Q Wang, **sp. nov.**, *Margattea paratransversa* J-J He & Z-Q Wang, **sp. nov.**, *Margattea disparilis* J-J He & Z-Q Wang, **sp. nov.**, *Margattea transversa* J-J He & Z-Q Wang, **sp. nov.**, and *Margattea bicruris* J-J He & Z-Q Wang, **sp. nov.**

## Introduction

Until now, 59 species have been included in the genus *Margattea* worldwide. Of these, 19 are from China (Wang et al. 2009; Liu et al. 2011; Beccaloni 2014). *Margattea* is known by the following characters: 1) eighth abdominal tergum unspecialized or specialized with a tuft; 2) median phallomere usually with accessory structure; 3) styli simple, cylindrical; and 4) symmetrical stripes and spots scattered on disc of pronotum, and in some species, the color of stripes and spots is similar to the body color (Roth 1989; Wang et al. 2009; Wang et al. 2014). As with other cockroach species, females of *Margattea* spp. are difficult to identify and match with males due to their strong resemblance in appearance and given that diagnostic characters are based on male genitalia (Wang et al. 2009, 2014).

DNA barcoding has proven to be a reliable and cost-effective method for identifying species in insect groups (Foster et al. 2004; Rach et al. 2008). General Mixed Yule-Coalescent (GMYC) (Pons et al. 2006), Automatic Barcode Gap Discovery (ABGD) (Puillandre et al. 2012), and Poisson-Tree-Processes (bPTP) (Zhang et al. 2013) have been used for species delimitation based on COI data (Che et al. 2017; Bai et al. 2018; Yang et al. 2019; Li et al. 2020).

In this study, we explore the diversity of *Margattea* species in China using both morphological features and GMYC, ABGD, and bPTP approaches to estimate the number of molecular operational taxonomic units (MOTUs), describe new species, and pair the female specimens with the males.

## Materials and methods

### Morphological study

Terminology mainly follows McKittrick (1964) (genitalia), Roth (2003), and Li et al. (2018) (venation). Venation abbreviations are as follows: cubitus anterior (CuA), cubitus posterior (CuP), media (M), radius (R), radius anterior (RA), radius posterior (RP), subcosta posterior (ScP), vannal (V), and postcubitus (Pcu).

Measurements are based on observed specimens. The genital segments of the studied specimens were dissected and immersed in 10% NaOH, heated to dissolve the fat, and rinsed with distilled water to make the segments and genitalia observable. They were then stored in glycerin. Genitalia were observed in glycerin using a MOTIC K400 stereomicroscope. All photos were made with a Leica DFC digital microscope camera attached to a Leica M205A stereomicroscope, and were modified with Adobe Photoshop CS6 (Adobe Systems, San Jose, CA, USA). Type materials are all deposited in the Institute of Entomology, Southwest University, Chongqing, China (SWU).

### DNA extraction, PCR and sequencing

DNA was extracted according to the Hipure Tissue DNA Mini Kit (Magen Biotech, Guangzhou). Fragments of COI were amplified using PCR. Primers used for the amplifications are LCO1490 (5'-GGTCAACAAATCATAAGATATTGG-3') and HCO2198 (5'-TAAACTTCAGGGTGACCAAAAAATCA-3') (Folmer et al. 1994). Each PCR was performed in Analytik Jena Easy Cycler with 25 μl volumes using the aforementioned primers, followed by agarose gel electrophoresis. Amplification conditions were: initial denaturation at 98 °C for 2 min, followed by 35 cycles for 10 s at 98 °C, 10 s at 49 °C, and 1 min at 72 °C, with a final extension of 3 min at 72 °C.

### Sequence processing and phylogenetic analyses

A total of 81 COI sequences were used for analysis, of which 68 sequences are newly sequenced and nine sequences were downloaded from GenBank. Four sequences were selected as outgroups from species of four genera (*Allacta*, *Sorineuchora*, *Balta*, and *Shelfordina*) of the subfamily Pseudophyllodromiinae (Table 1). All sequences were aligned using MEGA 7 and adjusted visually after translation into amino acid sequences, whose lengths were 658 bp. The genetic divergence value was quantified by MEGA 7 based on Kimura 2-parameter (K2P) (Kumar et al. 2016). Maximum likelihood (ML) analysis was implemented in RAxML 7.3.0 (Stamatakis et al. 2008) using a GTR GAMMA model with 1000 bootstrap replicates.

**Table 1.** Samples used in this study. The location numbers correspond to Figure 11.

Species	Voucher ID	GenBank accession number	Collecting information	Location number
**ingroups**				
*M. speciosa*		KY349620		
	M14_5	MW970279	Jianfengling, Hainan, China; date and collector unknown	1
*M. angusta*	M28_6	MW970280	Putian, Fujian, China; 21 July 2013; Shun-Hua Gui, Yan Shi	5
		KY349624		
*M. mckittrickae*	M29_1	MW970281	Baoting, Hainan, China; 2 May 2013; Shun-Hua Gui, Yan Shi	2
	M29_2	MW970282		
*M. spinifera*	M28_2	MW970272	Putian, Fujian, China; 21 July 2013; Shun-Hua Gui, Yan Shi	5
	M28_3	MW970273		
	M28_7	MW970277	Guiping, Guangxi, China; 31 May-2 June 2014; Shun-Hua Gui, Xin-Ran, Jian-Yue Qiu	19
	M28_8	MW970278		
	M28_9	MW970274	Fuzhou, Fujian, China; 26 July 2013; Yan Shi	6
	M28_10	MW970275	Mt Wuyi, Fujian, China; 6–30 July 2013; Shun-Hua Gui, Yan Shi	8
	M28_11	MW970276	Mt Taimu, Ningde City, Fujian, China; 6–30 July 2013; Shun-Hua Gui, Yan Shi	7
		KY349644		
*M. spinosa*	M30_7	MW970299	Baoting, Hainan, China; 2 May 2013; Yan Shi	2
		KY349617		
*M. bisignata*	M19_1	MW970312	Nanling, Guangdong, China; 5–7 June 2010; Collector Unkown.	16
	M19_2	MW970313		
	M19_3	MW970314		
	M19_4	MW970317	Mt E’mei, Sichuan, China; 2 June 2011; Ke-Liang Wu	24
	M19_5	MW970318		
	M19_6, F	MW970307	Guiping, Guangxi, China; 31 May–2 June 2014; Shun-Hua Gui, Xin-Ran, Jian-Yue Qiu	19
	M19_7, F	MW970308	Jingxiu, Guangxi, China; 4–5 June 2014; Shun-Hua Gui, Xin-Ran Li	18
	M19_8	MW970316	Mt Dabie, Hubei, China; 2 July 2014; Xin-Ran Li	12
	M19_9	MW970315	Beibei, Chongqing, China; 23 May 2013; Jin-Jin Wang	23
	M19_10	MW970319	Mt E’mei, Sichuan, China; 2 July 2013; Jin-JinWang, Yang Li	24
	M_SY	MW970309	Nanchang, Jiangxi, China; 3 June 2017; Xin-Ran Li, Li-Li Wang; Meng Li	14
	SP6_SY	MW970311	Mt Lu, Jiangxi, China; date and collector unknown	13
	SP6_SY_2	MW970310		
		KY349607		
*M. multipunctata*	M42_1	MW970271	Xishuangbanna, Yunnan, China; 17 November 2009; Guo Tang, Zhi-Yuan Yao.	26
	M_DB	MW970270	Xishuangbanna, Yunnan, China; 27 May 2016; Zhi-Wei Qiu, Lu Qiu.	26
	DB	MW970269		
		KY349646		
*M. nimbata*	M13_1	MW970258	Beibei, Chongqing, China; 15–19 June 2016, Yang Li	23
	M13_2	MW970257		
	M13_3	MW970259		
	M_N	MW970260	Beibei, Chongqing, China; 9 June 2018; Collector Unkown.	
		KY349658		
	M13_4	MW970261	Mt Zijin, Jiangsu, China; 6–7 July 2014; Xin-Ran Li, Jian-yue Qiu, Yan Shi	11
*M. concava*	M27_1	MW970254	Mt Jianfengling, Hainan, China; 6 May 2013; Shun-Hua Gui, Yan Shi.	1
	M27_3	MW970255		
	M_AY	MW970256	Mt Jianfengling, Hainan, China; 24 April 2015; Lu Qiu, Qi-Kun Bai.	
	M27_4, F	MW970252	Mt Wuzhi, Hainan, China; 6 May 2013; Shun-Hua Gui, Yan Shi	2
	M27_5	MW970253	Mt Diaoluo, Hainan, China; 8 May 2013; Shun-Hua Gui, Yan Shi	4
		MF136391		
*M. cuspidata* sp. nov.	SP5	MW970300	Mt Daming, Guangxi, China; 2 July 2015; Lu Qiu, Qi-Kun Bai	20
	SP5_2	MW970301		
*M. caudata* sp. nov.	SP7	MW970283	Pu’er, Yunnan, China; 20 May 2016; Lu Qiu, Zhi-Wei Qiu	27
	SP7_2	MW970284		
*M. caudata* sp. nov.	SP7_3	MW970285		
	C3, F	MW970287		
	C4, F	MW970288		
	C5, F	MW970289		
	M901	MW970286		
*M. disparilis* sp. nov.	M_SC	MW970290	Xishuangbanna, Yunnan, China; 23 May 2016; Lu Qiu, Zhi-Wei Qiu	26
	SP9	MW970292		
	SP10	MW970291		
*M. deltodonta* sp. nov.	SP3_2	MW970294	Pingbian, Yunnan, China; 15 May 2016; Lu Qiu, Zhi-Wei Qiu	25
	SP3_3	MW970295		
	SP8_SP3	MW970298		
	CY03	MW970297		
	SP3	MW970293		
	SP3_2_2	MW970296		
*M. bicruris* sp. nov.	SP2_2	MW970303	Xishuangbanna, Yunnan, China; 23 May 2016; Lu Qiu, Zhi-Wei Qiu	26
	SP2_3	MW970304		
	SP2_4	MW970305		
	SP2_2_2	MW970302		
	M2	MW970306		
*M. transversa* sp. nov.	M9	MW970264	Pu’er, Yunnan, China; 20 May 2016; Lu Qiu, Zhi-Wei Qiu	27
	M903	MW970265		
	M904	MW970266		
	C2, F	MW970267		
	C7, F	MW970268		
		KY349661		
*M. paratransversa* sp. nov.	SP1	MW970262	Pu’er, Yunnan, China; 20 May 2016; Lu Qiu, Zhi-Wei Qiu	27
	SP1_2	MW970263		
**outgroups**				
*Allacta ornata*		KY349665		
*Sorineuchora nigra*		MF612149		
*Balta notulata*		KX051740		
*Shelfordina volubilis*		KY349562		

F: after voucher number means female sample.

We used three molecular species delimitation methods (GMYC, ABGD, bPTP) to delimit *Margattea* species based on COI sequences. For GMYC, time-resolved gene trees were estimated in BEAST 1.8.1 (Drummond and Rambaut 2007) with the parameters as follows: the uncorrelated lognormal (UCLN) relaxed clock model, the mean clock rate fixed to 1, the UPGMA starting tree and the tree prior as constant-size coalescent. The single-threshold GMYC method was then applied to generate the ultrametric gene tree using the SPLITS package (Ezard et al. 2009; Team 2013). Ultimately, we compared the groups delimited with the one-species null model using a likelihood ratio test. For ABGD, we used the Jukes-Cantor (JC69) model with a relative gap width X = 1.0, the rest of the parameters are set by default. For bPTP, we uploaded the converted file of the ML tree into the web site (https://species.h-its.org) with the default setting to obtain the results.

## Results

### Morphological delimitation of *Margattea*

Herein seven new species, *Margattea deltodonta* J-J He & Z-Q Wang, sp. nov., *Margattea cuspidata* J-J He & Z-Q Wang, sp. nov., *Margattea caudata* J-J He & Z-Q Wang, sp. nov., *Margattea paratransversa* J-J He & Z-Q Wang, sp. nov., *Margattea disparilis* J-J He & Z-Q Wang, sp. nov., *Margattea transversa* J-J He & Z-Q Wang, sp. nov., and *Margattea bicruris* J-J He & Z-Q Wang, sp. nov. are established on the basis of morphological characters, including male genitalia, combined with molecular data. Species descriptions are provided below (Figs 4–10).

### Molecular analysis

**Figure 1.**
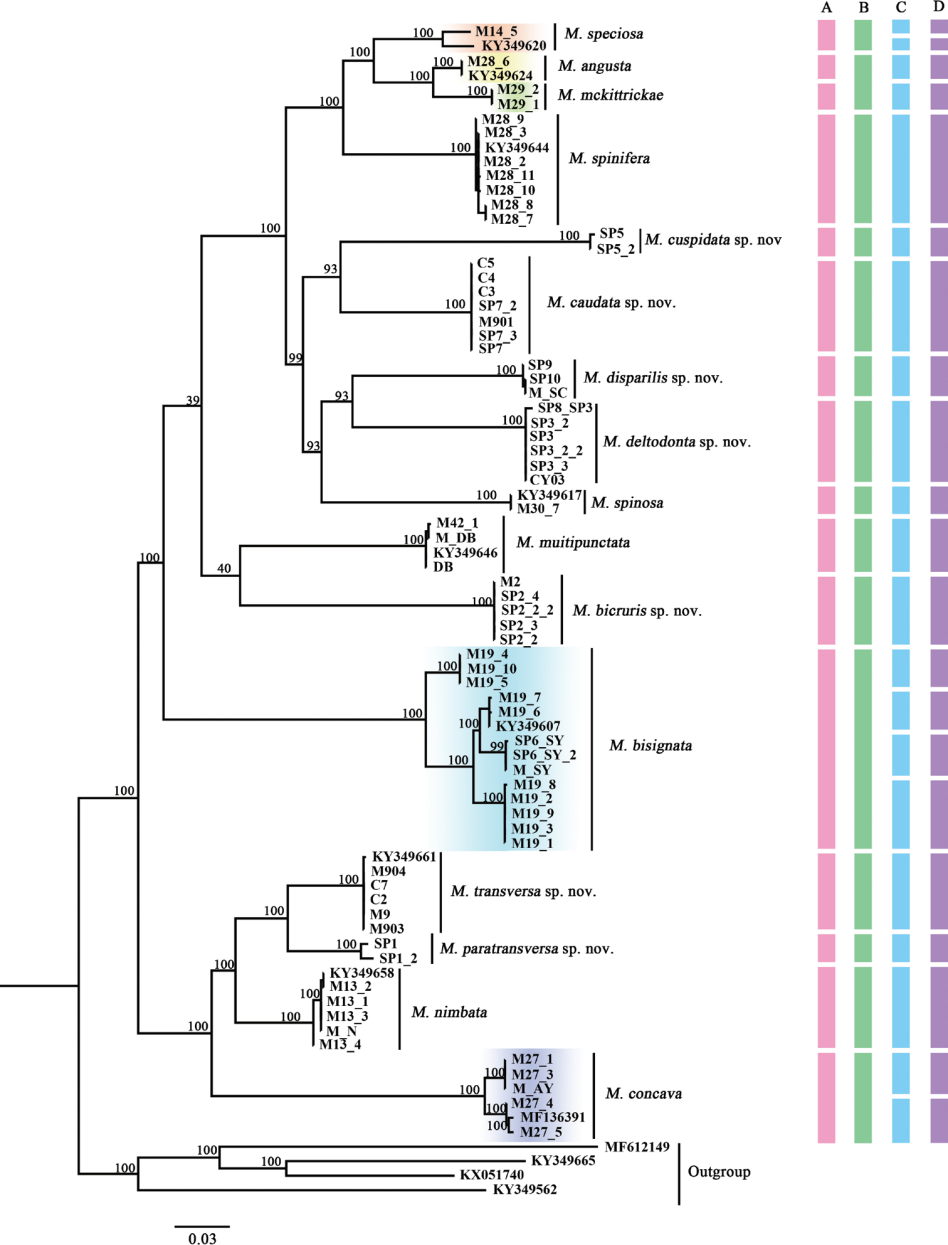
Maximum likelihood (ML) tree based on COI sequence. Branches labels is provided as bootstrap support values, some nodes without shown bootstrap value are given in Suppl. material 3: Fig. S1. Colored bars indicate different species delimitation by different methods **A** morphology (pink) **B**ABGD results (green) **C**GMYC results (blue) **D**bPTP results (purple). The colored clades on the tree (*M. speciosa*, *M. angusta*, *M. mckittrickae*, *M. bisignata*, and *M. concava*) correspond to clades with a disagreement between morphospecies and MOTUs.

All *Margattea* members were clustered together to form a monophyletic group in ML analysis (Fig. 1). Samples of *Margattea* species each formed monophyletic groups and most of branches with high support values, and females were recovered and grouped together with males (more details in Table 1). GMYC and bPTP analyses established 21 MOTUs as blue and purple bars indicate (Fig. 1); the ABGD analysis established 15 MOTUs (green bars). Compared to the other two molecular divisions, ABGD results were mostly consistent with morphological results (revealed by pink bars) (Fig. 1).

### Four methods to identify species

**Figure 2.**
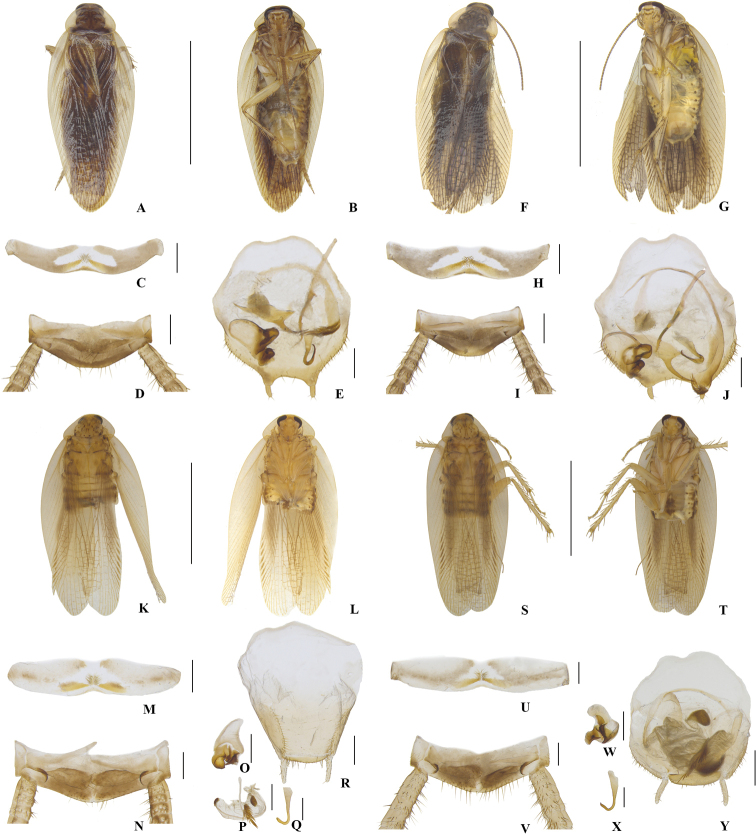
**A–J***Margattea concava***A–E** sample from Diaoluoshan, Hainan (voucher ID: M27_5), male **F–J** sample from Jianfengling, Hainan (voucher ID: M_AY), male **K–R***Margattea mckittrickae*, sample from Baoting, Hainan (voucher ID: M29_1), male **S–Y***Margattea angusta*, male **A, F, K, S** dorsal view **B, G, L, T** ventral view **C, H, M, U** eighth abdominal terga, ventral view **D, I, N, V** supra-anal plate and paraprocts, ventral view **E, J, R, Y** subgenital plate and phallomeres, dorsal view **O, W** left phallomere, dorsal view **P** median phallomere, dorsal view **Q, X** hook-like phallomere, dorsal view. Scale bars: 5 mm (**A–B, F–G, K–L, S–T**), 0.5 mm (**C–E, H–J, M–R, V–Y**).

**Figure 3.**
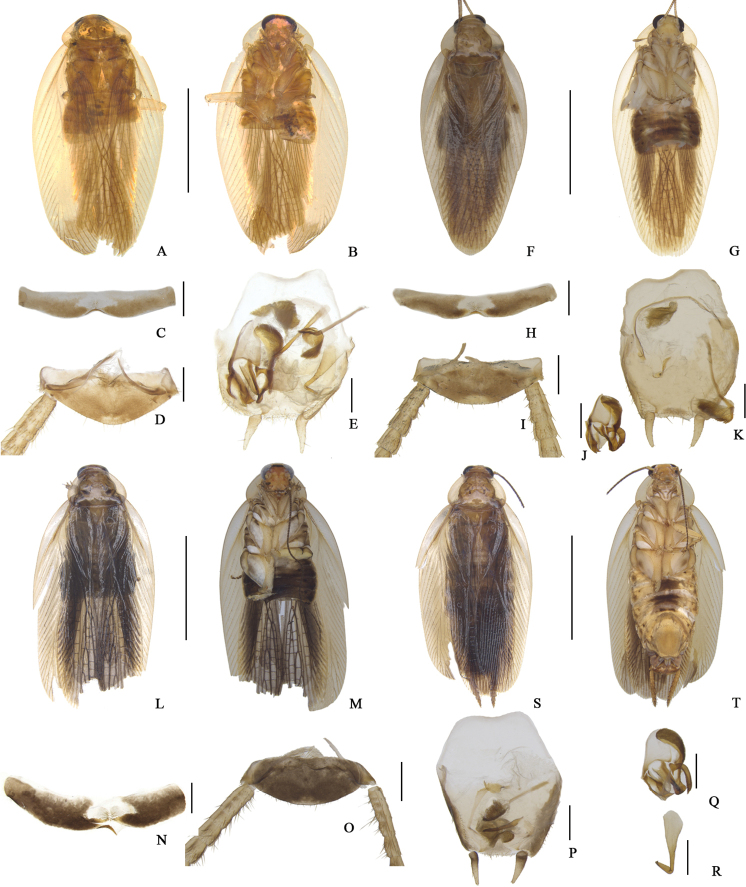
**A–R***Margattea bisignata***A–E** sample from E’meishan, Sichuan (voucher ID: M19_4), male **F–K** sample from Dabieshan, Hubei (voucher ID: M19_8), male **L–R** sample from Lushan, Jiangxi (voucher ID: SP6_SY), male **S–T** sample from Guiping, Guangxi (voucher ID: M19_6), female **A, F, L, S** dorsal view **B, G, M, T** ventral view **C, H, N** eighth abdominal terga, ventral view **D, I, O** supra-anal plate and paraprocts, ventral view **E, K, P** subgenital plate and phallomeres, dorsal view **J, Q** left phallomere, dorsal view **R** hook-like phallomere, dorsal view. Scale bars: 5 mm (**A–B, F–G, L–M, S–T**), 0.5 mm (**C–E, H–K, N–R**).

On the basis of morphological characters including male genitalia, we were able to identify 16 morphospecies of *Margattea*. ML analysis revealed each morphological species of the genus as a robust clade (Fig. 1). There were some similarities and differences in the results of these four methods. Both GMYC and bPTP divided all *Margattea* species into 21 MOTUs, while ABGD was different from the above two methods in that all species were divided into 15 MOTUs. And there were some disagreements between morphospecies and MOTUs, such as the colored clades on the ML tree. According to the GMYC and bPTP results, *M. speciosa* (with orange highlight) and *M. concava* (with lavender highlight) were grouped into two MOTUs. Moreover, *M. bisignata* (with light blue highlight) was divided into four MOTUs. And for ABGD, most species were consistent with morphospecies, except for *M. angusta* (with yellow highlight) and *M. mckittrickae* (with green highlight), which were considered to be one MOTU. As to the incongruence, we checked the specimens of *M. speciosa*, *M. concava*, and *M. bisignata* again and found there were no differences in male genitalia of their different samples (Figs 2, 3), so that the genetic variations among different samples of *M. speciosa*, *M. concava*, and *M. bisignata* were determined to be intraspecific differences despite a relatively large genetic distance existed (2.9% in *M. concava*, 3.1% in *M. speciosa*, and 5.9% in *M. bisignata*) (Suppl. material 1: Table S1). Also, upon examination of specimens of *M. angusta* and *M. mckittrickae*, we found some stable differences between the two species (in the former, the interstylar region barely protruding, right phallomere simple and hook-like, and in the latter, the interstylar region strongly produced, hook-like phallomere on the right side with a brush-shaped sclerite), although the genetic distance between them was only about 5%.

## Taxonomy

### Diagnosis of the genus *Margattea*

Third and fourth palpi both obviously longer than the fifth. Tegmina and wings usually fully developed, beyond end of abdomen, but slightly reduced in a few species, not reaching end of abdomen. Disc of pronotum usually with symmetrical maculae. ScP of tegmina simple, R multi-branched, M with 4–7 complete branches; hind wings of the ScP and RA expanded at base, CuA usually with 4–6 branches. Eighth abdominal tergum unspecialized or specialized with a tuft. Anteroventral margin of front femur type B_2_, or B_3_, rarely C_2_. Tarsal claws symmetrical and usually specialized, inner margin serrated. Styli simple, cylindrical. Hook phallomere on right. Median phallomere with accessory structure.

The genus *Margattea* Shelford, 1911 is closely related to *Balta* Tepper, 1893; however, they can be distinguished by the following characteristics: 1) In the former, the front femur is always of type B_2_, or B_3_, rarely C_2_; in the latter, the front femur always of type C_2_ or in a few, type B_3_; 2) in the former, the tarsal claws are symmetrical and specialized, but in the latter, the tarsal claws are asymmetrical and unspecialized; 3) in the former, the interstylar region is always convex or nearly straight, while in the latter, the interstylar region is always concave.

### Key to species of *Margattea* from China

**Table:** 

1	Tegmina basically reaching or extending beyond the end of abdomen	**2**
–	Tegmina barely reaching middle of abdomen	***M. hemiptera* Bey-Bienko, 1958**
2	The front femur Type B3	**3**
–	The front femur Type B2	**11**
3	Interstylar region have no produced or unconspicuous	**4**
–	Interstylar region strongly produced	**5**
4	Pronotum pale yellow without dark maculae	***M. immaculata* Liu & Zhou, 2011**
–	Pronotum yellowish brown with maculae	***M. mckittrickae* Wang, Che & Wang, 2009**
5	Interstylar region produced nearly rectangle-shaped	**6**
–	Interstylar region produced not rectangle-shaped	**7**
6	Posterior margin of interstylar region with a row of spines	***M. perspicillaris* (Karny, 1915)**
–	Posterior margin of interstylar region without spines	***Margattea angusta* Wang et al., 2014**
7	Interstylar region produced nearly arc-shaped with a row of spines	***M. spinifera* Bey-Bienko, 1958**
–	Interstylar region produced not arc-shaped	**8**
8	Interstylar region extremely asymmetrical	***M. disparilis* J-J He & Z-Q Wang, sp. nov.**
–	Interstylar region basically symmetrical	**9**
9	The left and right edges of interstylar region curl inward	**10**
–	The trailing edge of interstylar region curls upward	***M. furcata* Liu & Zhou, 2011**
10	The left end of the accessory structure of median phallomere with a slender bone	***M. cuspidata* J-J He & Z-Q Wang, sp. nov.**
–	The accessory structure of median phallomere without bones	***M. flexa* Wang et al., 2014**
11	Male eighth tergum unspecialized	**12**
–	Male eighth tergum specialized	**14**
12	Posterior margin of supra-anal plate with sharp protrusions	***M. producta* Wang, Che & Wang, 2009**
–	Posterior margin of supra-anal plate not produced and nearly straight	**13**
13	Ventral surface of body with brown spots	***M. punctulata* (Brunner von Wattenwyl, 1893)**
–	Ventral surface of body without brown spots	***M. limbata* Bey-Bienko, 1954**
14	Styli dissimilar, the left bigger than the right	***M. pseudolimbata* Wang et al., 2014**
–	Styli similar	**15**
15	Pronotal disc black brown with white maculae (specimens, the maculae of living body is fluorescent blue)	***M. multipunctata* Wang, Che & Wang, 2009**
–	Pronotal disc with scattered symmetrical maculae	**16**
16	The accessory structure of left phallomere with brush-shaped sclerite	***M. bisignata* Bey-Bienko, 1970**
–	The accessory structure of left phallomere without brush-shaped sclerite	**17**
17	Median phallomere with spinelike sclerites	**18**
–	Median phallomere without spinelike sclerites spinelike sclerites	**21**
18	Median phallomere with two or more spinelike sclerites	**19**
–	Median phallomere with only one spinelike sclerite	**20**
19	Median phallomere with three spinelike sclerite	***M. trispinosa* (Bey-Bienko, 1958)**
–	Median phallomere with two spinelike sclerite	***M. nimbata* (Shelford, 1907)**
20	Interocular space without brown band	***M. paratransversa* J-J He & Z-Q Wang, sp. nov.**
–	Interocular space with brown band	***M. transversa* J-J He & Z-Q Wang, sp. nov.**
21	Interstylar region strongly produced	**22**
–	Interstylar region have no produced or unconspicuous	**23**
22	Interstylar region convex fishtail-shaped	***M. caudata* J-J He & Z-Q Wang, sp. nov.**
–	Interstylar region convex triangular	***M. deltodonta* J-J He & Z-Q Wang, sp. nov.**
23	Interstylar region concave	***M. concava* Wang, Che & Wang, 2009**
–	Interstylar region not concave	**24**
24	Median phallomere with spines at apex	***M. spinosa* Wang et al., 2014**
–	Median phallomere without spines at apex	**25**
25	Median phallomere forked at apex	***M. bicruris* J-J He & Z-Q Wang, sp. nov.**
–	Median phallomere unforked	***M. speciosa* Liu & Zhou, 2011**

#### Margattea deltodonta

Taxon classificationAnimaliaBlattodeaEctobiidae

J-J He & Z-Q Wang sp. nov.

2D691CF1-26EB-5F1B-BD34-5B7A502C67D6

http://zoobank.org/34AE83CF-363C-4738-B42D-20AE048DC6C0

Figure 4A–N

##### Type material.

***Holotype*:** China • ♂; Hongqi Reservoir, Mt Dawei, Pingbian County, Yunnan Province; 1550 m, 15-V-2016; Lu Qiu, Zhi-Wei Qiu leg; SWU-B-EC141501. ***Paratypes***: China • 3♂♂; same data as holotype; SWU-B-EC141502-141504.

##### Other material.

China • 1♂; Hongqi Reservoir, Mt Dawei, Pingbian County, Yunnan Province; 1550 m; 17-V-2016; Lu Qiu, Zhi-Wei Qiu leg.

##### Diagnosis.

This species is similar to *M. satsumana* (Asahina, 1979) in general appearance, but can be differentiated from the latter by the following characters: 1) median phallomere slender rod with base sharp, and apex expanded with three spines; while in the latter, base slightly expanded, and apex curved with some short spines; 2) subgenital plate not folded; while in the latter, folded inwards.

##### Measurements

**(mm).** Male (*n* = 4), pronotum: length × width 1.6–2.1 × 2.6–2.9, tegmina length: 10.3–11.2, overall length: 12.5–13.1.

##### Description.

**Figure 4.**
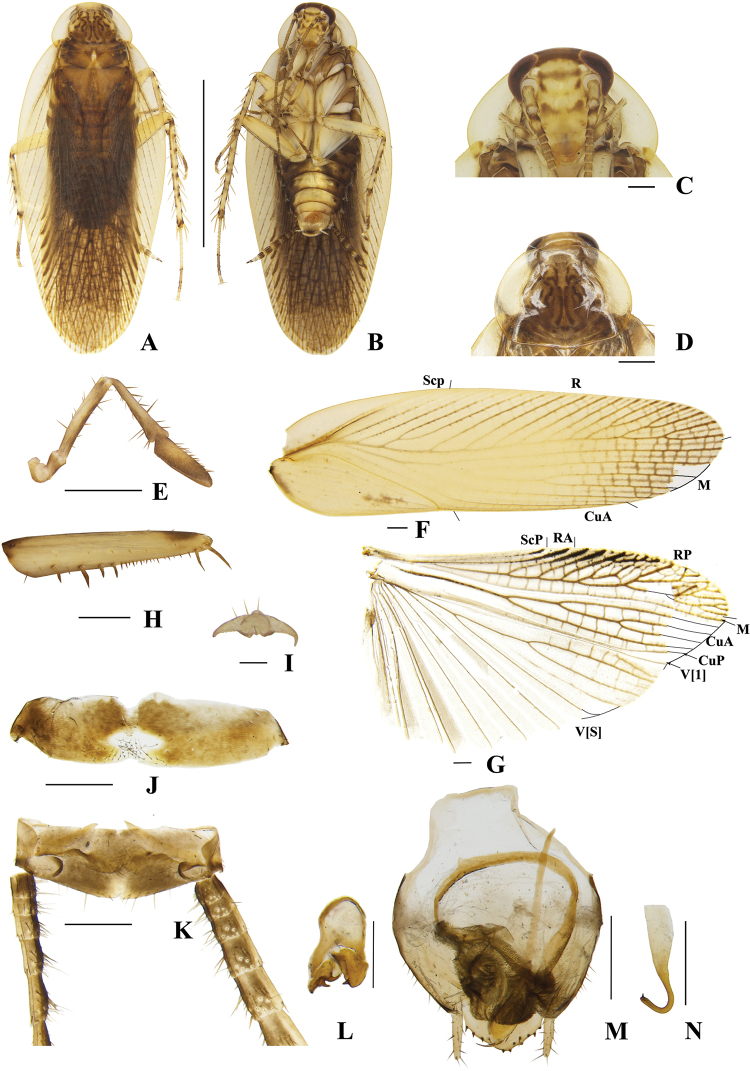
**A–N***Margattea deltodonta* sp. nov., male **A** holotype, dorsal view **B** holotype, ventral view **C** head, ventral view **D** pronotum, dorsal view **E** maxillary palpi, ventral view **F** tegmen, dorsal view **G** hind wing, dorsal view **H** front femur, ventral view **I** tarsal claws **J** eighth abdominal terga **K** supra-anal plate and paraprocts, ventral view **L** left phallomere, dorsal view **M** subgenital plate and median phallomere, dorsal view **N** hook-like phallomere, dorsal view. Scale bars: 5 mm (**A, B**); 0.5 mm (**C–H, J, K–N**); 0.1 mm (**I**).

**Male. *Coloration***: body yellowish-brown (Fig. 4A, B). Face pale yellowish-brown. Interocular space with a dark brown band. Ocelli spots white, interocellar space with a brown band. Antennae dark linen. Clypeus medium yellowish-brown (Fig. 4C). Maxillary palps dark yellowish-brown (Fig. 4E). Pronotal disc yellowish-brown with brown stripes and spots, and two lateral borders light brown (Fig. 4D). Tegmina medium brown, wings light linen (Fig. 4F, G). Abdomen yellowish-brown (Fig. 4B). Cerci dark yellowish-brown (Fig. 4K). Styli faint yellow (Fig. 4M). ***Head***: vertex slightly exposed, interocular distance same length as antennal sockets space (Fig. 4C). Pronotum nearly trapezoidal, broader than long, the widest part after the midpoint, the front and posterior margins nearly straight, and the postero-lateral angle blunt and round; the disc with symmetrical irregular macules (Fig. 4D). The third and fourth palpi about same length, both obviously longer than the fifth, the fifth obviously expanded (Fig. 4E). ***Tegmina and wings***: tegmina and wings fully developed, both extending beyond the end of abdomen (Fig. 4A, B). Tegmina with ScP simple, R multi-branched, M straight with five complete branches. Hind wings with ScP and RA expanded at apex; M straight and simple, without branches; CuA with four complete branches (Fig. 4F, G). ***Legs***: anteroventral margin of front femur type B_2_ (Fig. 4H). Pulvilli present on four proximal tarsomeres. Tarsal claws symmetrical and specialized, inner margin serrated, arolia present (Fig. 4I). ***Abdomen and genitalia***: eighth abdominal tergum specialized with a tuft (Fig. 4J). Supra-anal plate transverse, posterior margin convex, the middle slightly concave. Paraprocts simple, similar, splitting into two pieces, apex with tufts (Fig. 4K). Subgenital plate asymmetrical, both lateral margins slightly concave. Styli similar, slender with spines; interstylar region obviously convex with small spines (Fig. 4M). Left phallomere complex, irregular bone-shaped, with two short spines (Fig. 4L). Median phallomere slender rod-shaped with base sharp, and apex expanded with three long spines; the accessory structure arched, at rightmost end brush-shaped (Fig. 4M). Hook phallomere on the right side, apex curved inwards with a short spine (Fig. 4N).

**Female** unknown.

##### Etymology.

The word “delt” and “odont” from Greek and means triangular, the species name “*deltodontus*” refers to the posterior margin of subgenital plate with small spines.

##### Distribution.

China (Yunnan).

#### Margattea cuspidata

Taxon classificationAnimaliaBlattodeaEctobiidae

J-J He & Z-Q Wang sp. nov.

6555CE28-87CB-5E75-8E02-A54A2E2C27E0

http://zoobank.org/7AD3ADF0-DA60-493B-A229-363CBC71F002

Figure 5A–N

##### Type material.

***Holotype*:** China • ♂; Mt Daming, Guangxi Province; 2-VII-2015; Lu Qiu, Qi-Kun Bai leg; SWU-B-EC141201. ***Paratype***: China • 1♂; same data as for holotype; SWU-B-EC141202.

##### Diagnosis.

This species is similar to *M. flexa*Wang et al., 2014 in general appearance and male genitalia, but it can be differentiated from the latter by the following characters: 1) interstylar region obviously convex with both sides curved inwards, three spines on each side, while in the latter, two sides curled up with 5–6 small thorns; 2) the left end of the accessory structure with a slender bone; the latter absent.

##### Measurements

**(mm).** Male (*n* = 2), pronotum: length × width 1.6–2.1 × 2.6–2.9, tegmina length: 10.3–11.2, overall length: 12.5–13.1.

##### Description.

**Figure 5.**
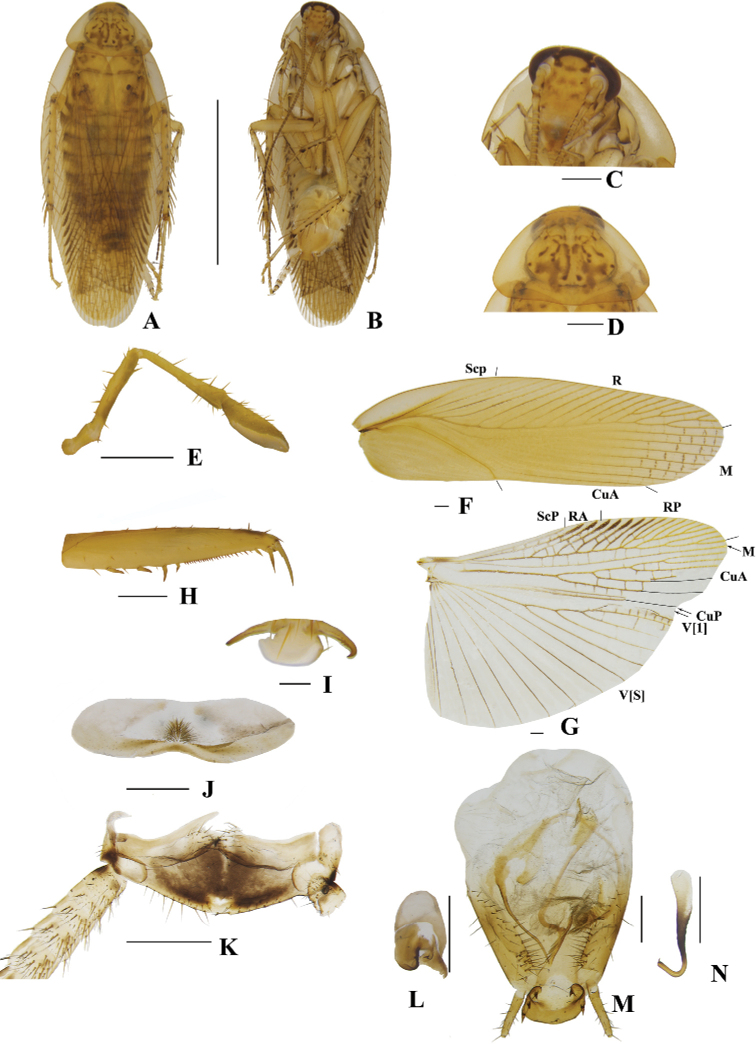
**A–N***Margattea cuspidata* sp. nov., male **A** holotype, dorsal view **B** holotype, ventral view **C** head, ventral view **D** pronotum, dorsal view **E** maxillary palpi, ventral view **F** tegmen, dorsal view **G** hind wing, dorsal view **H** front femur, ventral view **I** tarsal claws **J** eighth abdominal terga **K** supra-anal plate and paraprocts, ventral view **L** left phallomere, dorsal view **M** subgenital plate and median phallomere, dorsal view **N** hook-like phallomere, dorsal view. Scale bars: 5 mm (**A, B**); 0.5 mm (**C–H, J, K–N**); 0.1 mm (**I**).

**Male. *Coloration***: body yellowish-brown (Fig. 5A, B). Face yellowish-brown. Interocular space with a dark brown band. Ocelli spots white, interocelli space with a brown band. Antennae dark linen-colored. Clypeus dark yellowish-brown (Fig. 5C). Maxillary palps yellowish-brown (Fig. 5E). Pronotal disc yellowish-brown with dark brown stripes, and lateral borders light linen-colored (Fig. 5D). Tegmina pale yellow, wings medium brown (Fig. 5F, G). Abdomen pale yellowish-brown. Cerci pale yellowish-brown (Fig. 5K). Styli faint yellow (Fig. 5M). ***Head***: vertex slightly exposed, distance between interocular same length as antennal sockets space (Fig. 5C). Pronotum nearly trapezoidal, broader than long, the widest part after the midpoint, the front and posterior margins nearly straight, and the postero-lateral angle blunt and round; the disc with symmetrical irregular macules (Fig. 5D). The third, fourth palpi of approximately same length, both obviously longer than the fifth, the fifth obviously expanded (Fig. 5E). ***Tegmina and wings***: tegmina and wings fully developed, both extending beyond the end of abdomen (Fig. 5A, B). Tegmina with ScP simple, R multi-branched, M straight with six complete branches. Hind wings with ScP and RA expanded at apex; M straight and simple, without branches; CuA with five complete branches (Fig. 5F, G). ***Legs***: anteroventral margin of front femur type B_3_ (Fig. 5H). Pulvilli present on four proximal tarsomeres. Tarsal claws symmetrical and specialized, inner margin serrated, arolia present (Fig. 5I). ***Abdomen and genitalia***: eighth abdominal tergum specialized with a tuft (Fig. 5J). Supra-anal plate transverse, posterior margin protruded. Paraprocts similar, splitting into two pieces, apex with tufts (Fig. 5K). Subgenital plate symmetrical, lateral borders flip inwards with spines and hairs. Styli similar, slender; interstylar region obviously convex, two sides convex and curved inwards, each side with three spines (Fig. 5M). Left phallomere complex, irregular bone-shaped, with a short spine (Fig. 5L). Median phallomere slender rod-shaped, obviously curved, apex with ordered long spines; the accessory structure arched, on at rightmost end with spines, left apex with a slender bone with apex sharp (Fig. 5M). Hook phallomere on the right side, apex curved inwards with a short spine (Fig. 5N).

**Female** unknown.

##### Etymology.

The latin name “*cuspidatus*” refers to interstylar region obviously convex, two sides convex and curved inwards.

##### Distribution.

China (Guangxi).

#### Margattea caudata

Taxon classificationAnimaliaBlattodeaEctobiidae

J-J He & Z-Q Wang sp. nov.

43B31FEC-D167-5976-B8FC-C56AAA8EBF4D

http://zoobank.org/D3FF2635-DB1C-42D1-B087-890314430081

Figure 6A–N

##### Type material.

***Holotype*:** China • ♂; Meizihu Reservoir, Pu’er City, Yunnan Province; 1400 m; 21-V-2016; Lu Qiu, Zhi-Wei Qiu leg; SWU-B-EC141301. ***Paratypes***: China • 6♂♂; 1 ♀, same data as holotype SWU-B-EC141302-141308.

##### Other materials.

China • 2♀♀; Meizihu Reservoir, Pu’er City, Yunnan Province; 1400 m; 20-V-2016; Lu Qiu, Zhi-Wei Qiu leg.

##### Diagnosis.

This species is similar to *M. mckittrickae* Wang, Che & Wang, 2009 in general appearance, but it can be differentiated from the latter by the following characters: 1) interstylar region obviously convex, fishtail-shaped, while the latter slightly convex; 2) left phallomere complex, irregular bone-shaped, while in the latter, two sides of left phallomere sheet-like; and 3) median phallomere with one accessory structure, while the latter with two accessory structures.

##### Measurements

**(mm).** Male (*n* = 4), pronotum: length × width 2.7–2.8 × 3.1–3.6, tegmina length: 10.4–12.6, overall length: 12.8–14.1. Female, pronotum: length × width 2.5–3.0 × 3.6–3.7, tegmina length: 9.0–9.1, overall length: 11.7–12.6.

##### Description.

**Figure 6.**
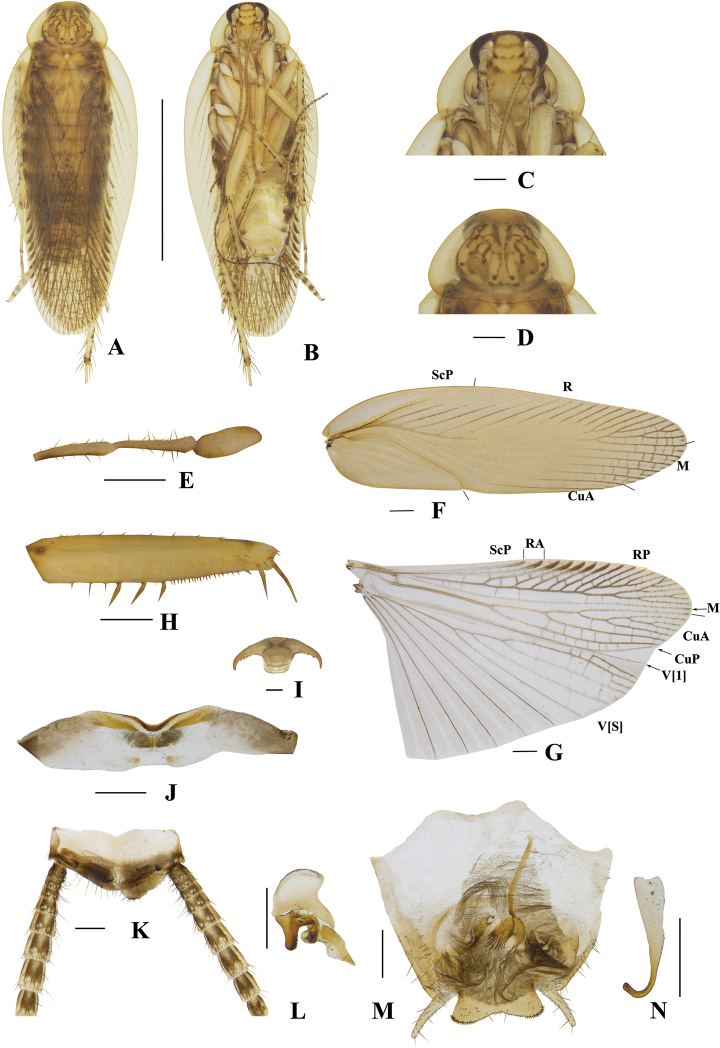
**A–N***Margattea caudata* sp. nov., male **A** holotype, dorsal view **B** holotype, ventral view **C** head, ventral view **D** pronotum, dorsal view **E** maxillary palpi segments 3–5, ventral view **F** tegmen, dorsal view **G** hind wing, dorsal view **H** front femur, ventral view **I** tarsal claws **J** eighth abdominal terga **K** supra-anal plate and paraprocts, ventral view **L** left phallomere, dorsal view **M** subgenital plate and median phallomere, dorsal view **N** hook-like phallomere, dorsal view. Scale bars: 5 mm (**A, B**); 0.5 mm (**C–H, J, K–N**); 0.1 mm (**I**).

**Male. *Coloration***: body pale brown with yellowish-brown (Fig. 6A, B). Face pale yellowish-brown. Interocular space with a brown band. Ocelli spots white, interocellar space with a brown band. Antennae light linen-colored. Clypeus medium yellowish-brown (Fig. 6C). Maxillary palps dark yellowish-brown (Fig. 6E). Pronotal disc pale yellowish-brown with brown stripes and two lateral borders light yellowish-brown (Fig. 6D). Tegmina yellowish-brown and wings medium brown (Fig. 6F, G). Abdomen pale yellowish-brown. Cerci yellowish-brown (Fig. 6K). Styli faint yellow (Fig. 6M). ***Head***: vertex slightly exposed, interocular distance interocular same length as antennal socket space (Fig. 6C). Pronotum nearly trapezoidal, broader than long, the widest part after the midpoint, the front and posterior margins nearly straight, and postero-lateral angle blunt and round; disc with symmetrical irregular macules (Fig. 6D). Third and fourth palpi of approximately same length, both obviously longer than the fifth, fifth palp obviously expanded (Fig. 6E). ***Tegmina and wings***: tegmina and wings fully developed, both extending beyond the end of abdomen (Fig. 6A, B). Tegmina with ScP simple, R multi-branched, M straight with five complete branches. Hind wings with ScP and RA expanded at apex; M straight and simple, without branches; CuA with 7 complete branches (Fig. 6F, G). ***Legs***: anteroventral margin of front femur type B_2_ (Fig. 6H). Pulvilli present on four proximal tarsomeres. Tarsal claws symmetrical and specialized, inner margin serrated, arolia present (Fig. 6I). ***Abdomen and genitalia***: eighth abdominal tergum specialized with a tuft (Fig. 6J). Supra-anal plate transverse, posterior margin protruded. Paraprocts similar, splitting into two pieces, apex with tufts (Fig. 6K). Subgenital plate symmetrical, the middle of front margin slightly concave; the base of two lateral margins concave, apex flips inwards with tufts. Styli similar, slender, distinctly separated; interstylar region obviously convex, fishtail-shaped, middle space slightly concave, two lateral angles convex with short spines (Fig. 6M). Left phallomere complex, irregular bone-shaped, with short spine (Fig. 6L). Median phallomere slender rod-shaped, apex splitting into two parts, each with some long spines (Fig. 6M). Hook phallomere on the right side, apex curved inwards with a short spine (Fig. 6N).

**Female** with tegmina and wings slightly reduced.

##### Etymology.

The latin name “*caudatus*” meaning “tail”, refers to the fishtail-shaped convexity on interstylar region.

##### Distribution.

China (Yunnan).

#### Margattea disparilis

Taxon classificationAnimaliaBlattodeaEctobiidae

J-J He & Z-Q Wang sp. nov.

1F87B345-5C95-56EF-8E4C-38A4256A6551

http://zoobank.org/484B73A3-9A9D-4922-81FB-CF7CE1E7C986

Figure 7A–N

##### Type material.

***Holotype*:** China • ♂; Wangtianshu Scenery Spot, Mengla County, Xishuangbanna Prefecture, Yunnan Province; 720 m; 23-V-2016; Lu Qiu, Zhi-Wei Qiu leg; SWU-B-EC141401. ***Paratypes***: China • ♂; Gougu Tropical Rainforest, Xishuangbanna Tropical Botanical Garden (CAS), Menglun Town, Jinghong City, Yunnan Province; 570 m; 26-V-2016; Lu Qiu, Zhi-Wei Qiu leg; SWU-B-EC141402.

##### Other material.

China • 1♂; Lvshilin (Green Stone Forest), Xishuangbanna Tropical Botanical Garden (CAS), Menglun Town, Jinghong City, Yunnan Province; 25-V-2016; Lu Qiu, Zhi-Wei Qiu leg.

##### Diagnosis.

This species is similar to *M. flexa*Wang et al., 2014 in male genitalia, but it can be differentiated from the latter by the following characters: 1) interstylar region obviously irregularly convex, the left part obviously larger than the right, while in the latter, interstylar region obviously regularly convex; 2) the left part of the accessory structure of median phallomere with a brush, absent in the latter.

##### Measurements

**(mm).** Male (*n* = 3), pronotum: length × width 2.4–2.6 × 3.2–3.5, tegmina length: 9.3–9.9, overall length: 11.2–11.9.

##### Description.

**Figure 7.**
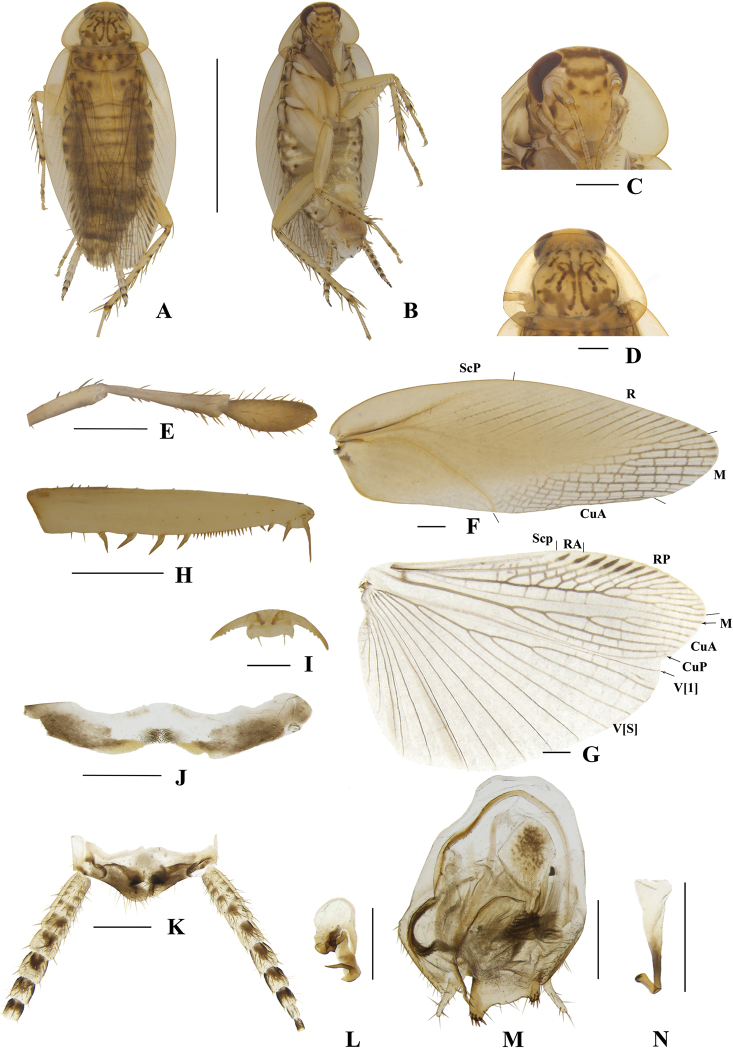
**A–N***Margattea disparilis* sp. nov., male **A** holotype, dorsal view **B** holotype, ventral view **C** head, ventral view **D** pronotum, dorsal view **E** maxillary palpi segments 3–5, ventral view **F** tegmen, dorsal view **G** hind wing, dorsal view **H** front femur, ventral view **I** tarsal claws **J** eighth abdominal terga **K** supra-anal plate and paraprocts, ventral view **L** left phallomere, dorsal view **M** subgenital plate and median phallomere, dorsal view **N** hook-like phallomere, dorsal view. Scale bars: 5 mm (**A, B**); 0.5 mm (**C–H, J, K–N**); 0.1 mm (**I**).

**Male. *Coloration***: body yellowish-brown with pale brown (Fig. 7A, B). Face yellowish-brown. Interocular space with a dark brown band. Ocellar spots white and small, interocellar space with a brown band. Antennae light linen- colored. Clypeus medium yellowish-brown (Fig. 7C). Maxillary palps light yellowish-brown to yellowish-brown (Fig. 7E). Pronotal disc yellowish-brown with brown stripes and two lateral borders light yellow (Fig. 7D). Tegmina light fawn, wings and legs pale brown (Fig. 7F, G). Abdomen light linen with pale yellowish-brown. Cerci pale brown (Fig. 7K). Styli light yellowish-brown (Fig. 7M). ***Head***: vertex slightly exposed, distance between interocular same length antennal sockets space (Fig. 7C). Pronotum nearly trapezoidal, broader than long, the widest part after the midpoint, the front and posterior margins nearly straight, and postero-lateral angle blunt and round; disc with symmetrical irregular macules (Fig. 7D). Third and fourth palpi of approximately the same length, both obviously longer than fifth, fifth palp obviously expanded (Fig. 7E). ***Tegmina and wings***: tegmina and wings developed, both extending the end of abdomen (Fig. 7A-B). Tegmina with ScP simple, R multi-branched, M straight with seven complete branches. Hind wings with ScP and RA expanded at apex; M straight and simple, without branches; CuA with five complete branches (Fig. 7F, G). ***Legs***: anteroventral margin of front femur type B_3_ (Fig. 7H). Pulvilli present on four proximal tarsomeres. Tarsal claws symmetrical and specialized, inner margin serrated, arolia present (Fig. 7I). ***Abdomen and genitalia***: eighth abdominal tergum specialized with a tuft (Fig. 7J). Supra-anal plate transverse, posterior margin convex. Paraprocts simple, similar, splitting into two pieces, base with tufts (Fig. 7K). Subgenital plate asymmetrical. Styli similar, slender, distinctly separated; interstylar region obviously irregularly convex, middle part concave, two lateral angles spherical with some short thorns, left angle obviously larger than right (Fig. 7M). Left phallomere complex, irregular bone-shaped, with a long spine (Fig. 7L). Median phallomere slender rod-shaped, base splitting into some long spines; the accessory structure arched, at leftmost end with a brush (Fig. 7M). Hook phallomere on right side, base curved inwards with a short spine (Fig. 7N).

**Female** unknown.

##### Etymology.

The latin name “*disparilis*” refers to the interstylar region obviously irregular convex.

##### Distribution.

China (Yunnan).

#### Margattea transversa

Taxon classificationAnimaliaBlattodeaEctobiidae

J-J He & Z-Q Wang sp. nov.

EA22E908-4A50-5924-A0AB-7551CAF6095C

http://zoobank.org/CA538705-BF0B-46C7-9708-29697F9F2ADD

Figure 8A–N

##### Type material.

**Holotype**: China • ♂; Meizihu Reservoir, Pu’er City, Yunnan Province; 20-V-2016; Lu Qiu, Zhi-Wei Qiu leg; SWU-B-EC141801. **Paratypes**: China • 2♂; same date as for holotype SWU-B-EC141802-141803.

##### Other materials.

China • 1♀; Meizihu Reservoir, Pu’er City, Yunnan Province; 20-V-2016; Lu Qiu, Zhi-Wei Qiu leg. • 1♀; Meizihu Reservoir, Pu’er City, Yunnan Province; 21-V-2016; Lu Qiu, Zhi-Wei Qiu leg.

##### Diagnosis.

This species is similar to *M. nimbata* (Shelford, 1907) in male genitalia, but it can be differentiated from the latter by the following characters: 1) median phallomere base with a curved spine, while in the latter, with two curved spines; 2) a long piece of bone extends from the right side of the accessory structure, while absent in the latter; and 3) left phallomere with four long spines; the latter with two long spines.

##### Measurements

**(mm).** Male (*n* = 3), pronotum: length × width 2.5–2.6 × 3.2–3.9, tegmina length: 11.7–12.3, overall length: 14.0–14.1. Female, pronotum: length × width 2.6–2.7 × 3.2–3.4, tegmina length: 11.3–11.5, overall length: 13.4–13.6.

##### Description.

**Figure 8.**
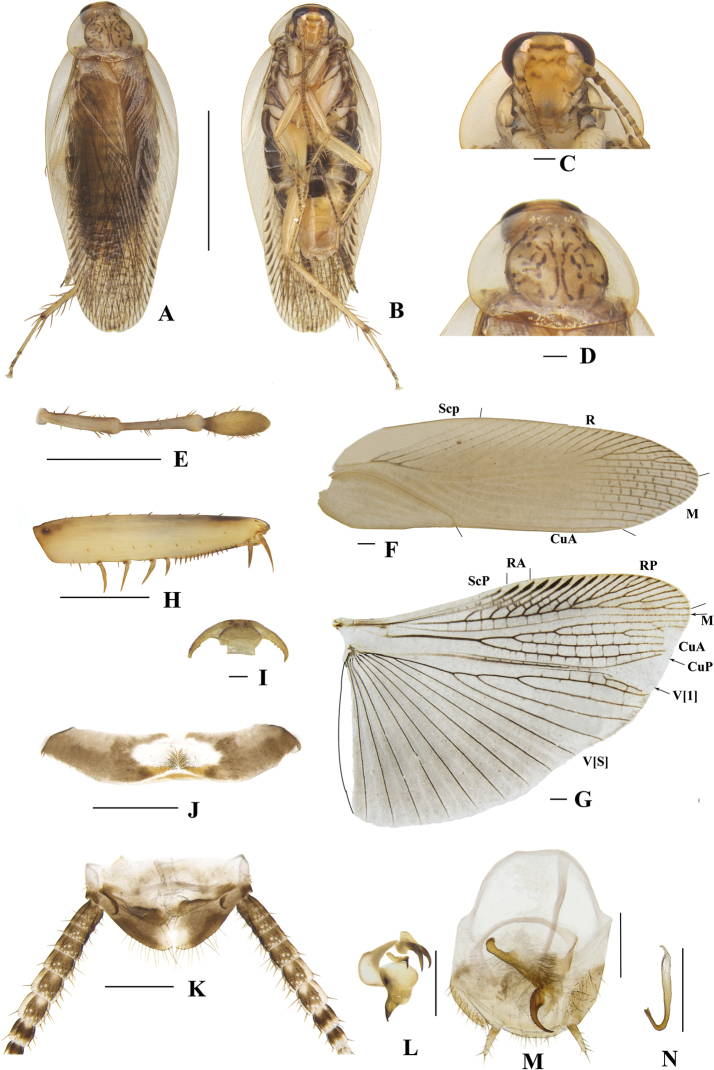
**A–N***Margattea transversa* sp. nov., male **A** holotype, dorsal view **B** holotype, ventral view **C** head, ventral view **D** pronotum, dorsal view **E** maxillary palpi segments 3–5, ventral view **F** tegmen, dorsal view **G** hind wing, dorsal view **H** front femur, ventral view **I** tarsal claws **J** eighth abdominal terga **K** supra-anal plate and paraprocts, ventral view **L** left phallomere, dorsal view **M** subgenital plate and median phallomere, dorsal view **N** hook-like phallomere, dorsal view. Scale bars: 5 mm (**A, B**); 0.5 mm (**C–H, J, K–N**); 0.1 mm (**I**).

**Male. *Coloration***: body pale yellowish-brown with brown (Fig. 8A, B). Face yellowish-brown. Interocular space with a dark brown band. Ocelli spots white and big, interocelli space with a brown band. Antennae pale yellowish-brown. Clypeus medium yellowish-brown. Maxillary palps light yellowish-brown (Fig. 8E). Pronotal disc brownish-gray with brown stripes and two lateral borders light grey (Fig. 8D). Tegmina pale yellowish-brown, wings grey brown (Fig. 8F, G). Abdomen yellowish-brown. Cerci pale yellowish-brown to pale brown (Fig. 8K). Styli faint yellow (Fig. 8M). ***Head***: vertex slightly exposed, interocular distance same length as antennal socket space (Fig. 8C). Pronotum nearly trapezoidal, broader than long, widest part after midpoint, front and posterior margins nearly straight, and postero-lateral angle blunt and round; disc with symmetrical irregular macules (Fig. 8D). Third and fourth palpi of approximately same length, both obviously longer than fifth palp, fifth palp obviously expanded (Fig. 8E). ***Tegmina and wings***: tegmina and wings fully developed, both extending beyond the end of abdomen (Fig. 8A, B). Tegmina with ScP simple, R multi-branched, M straight with seven complete branches. Hind wings with ScP and RA expanded at base; M straight and simple, without branches; CuA with five complete branches (Fig. 8F, G). ***Legs***: anteroventral margin of front femur type B_2_ (Fig. 8H). Pulvilli present on four proximal tarsomeres. Tarsal claws symmetrical and specialized, inner margin serrated, arolia present (Fig. 8I). ***Abdomen and genitalia***: eighth abdominal tergum specialized with a tuft (Fig. 8J). Supra-anal plate transverse, posterior margin convex. Paraprocts simple, similar, splitting into two pieces (Fig. 8K). Subgenital plate symmetrical. Styli similar, slender, distinctly separated (Fig. 8M). Left phallomere complex, irregular bone-shaped, with four spines (Fig. 8L). Median phallomere slender rod-shaped, apex with a curved spine; the accessory structure arched, at rightmost end blunt (Fig. 8M). Hook phallomere on the right side, base curved inwards with a short spine (Fig. 8N).

**Female** same as male.

##### Etymology.

The latin name “*transversus*” refers to the interocular space having a dark brown transverse band.

##### Distribution.

China (Yunnan).

#### Margattea paratransversa

Taxon classificationAnimaliaBlattodeaEctobiidae

J-J He & Z-Q Wang sp. nov.

8E1E1D5B-5D7E-57F0-B4A0-768CE9D396BF

http://zoobank.org/268B5D3F-BC3D-4A0B-93B7-6DC75B28FB60

Figure 9A–N

##### Type material.

***Holotype*:** China • ♂; Meizihu Reservoir, Pu’er City, Yunnan Province; 1400 m; 21-V-2016; Lu Qiu, Zhi-Wei Qiu leg; SWU-B-EC141701. ***Paratype***: China • 6♂♂; same data as holotype; SWU-B-EC141702-141707.

##### Other material.

China • 2♀; Meizihu Reservoir, Pu’er City, Yunnan Province; 1400 m; 20-V-2016; Lu Qiu, Zhi-Wei Qiu leg.

##### Diagnosis.

This species closely resembles *Margattea transversa* sp. nov., but they can be distinguished by the following characteristics: 1) Left phallomere of the former with three long spines, while the latter with four long spines; 2) In the former, median phallomere apex with a slightly curved spine, while the median phallomere apex of latter with a distinct curved spine. In addition, this species is also similar to *M. nimbata* (Shelford, 1907) in general appearance, but it can be differentiated from the latter by the following characters: 1) median phallomere base with a curved spine, while in the latter, with two curved spines; 2) A long piece of bone extends from the right side of the accessory structure, while absent in the latter; and 3) left phallomere with three long spines; the latter with two long spines.

##### Measurements

**(mm).** Male (*n* = 5), pronotum: length × width 2.7–2.8 × 3.1–3.6, tegmina length: 10.4–12.6, overall length: 12.8–14.1.

##### Description.

**Figure 9.**
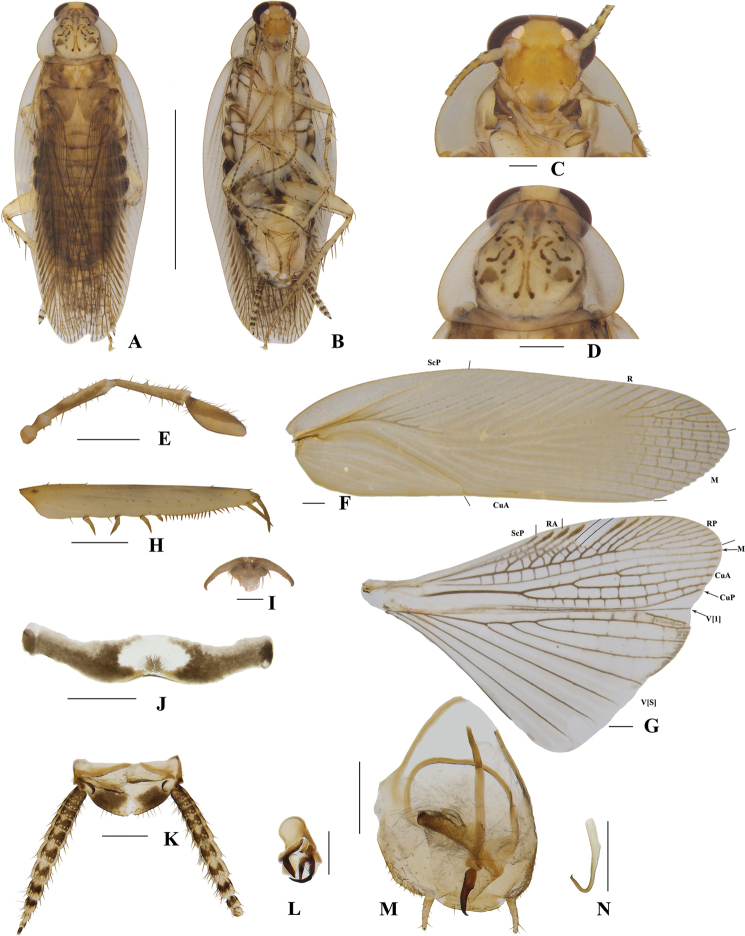
**A–N***Margattea paratransversa* sp. nov., male **A** holotype, dorsal view **B** holotype, ventral view **C** head, ventral view **D** pronotum, dorsal view **E** maxillary palpi, ventral view **F** tegmen, dorsal view **G** hind wing, dorsal view **H** front femur, ventral view **I** tarsal claws **J** eighth abdominal terga **K** supra-anal plate and paraprocts, ventral view **L** left phallomere, dorsal view **M** subgenital plate and median phallomere, dorsal view **N** hook-like phallomere, dorsal view. Scale bars: 5 mm (**A, B**); 0.5 mm (**C–H, J, K–N**); 0.1 mm (**I**).

**Male. *Coloration***: body pale yellowish-brown with yellowish-brown (Fig. 9A, B). Face dark yellowish-brown. Interocular space with a brown band. Ocelli spots white. Antennae yellowish-brown. Clypeus pale brown (Fig. 9C). Maxillary palps light linen-colored (Fig. 9E). Pronotal disc light linen-colored with brown stripes and two lateral borders yellowish-white (Fig. 9D). Tegmina pale yellowish-brown, wings medium brown (Fig. 9F, G). Abdomen cream-colored to pale brown. Cerci yellowish brown (Fig. 9K). Styli faint yellow (Fig. 9M). ***Head***: vertex slightly exposed, distance between interocular shorter than antennal socket space (Fig. 9C). Pronotum nearly trapezoidal, broader than long, the widest part after the midpoint, the front and posterior margins nearly straight, and the postero-lateral angle blunt and round; disc with symmetrical irregular maculae (Fig. 9D). Third and fourth palpi of approximately same length, both obviously longer than fifth palp, fifth palp obviously expanded (Fig. 9E). ***Tegmina and wings***: tegmina and wings fully developed, both extending beyond the end of abdomen (Fig. 9A, B). Tegmina with ScP simple, R multi-branched, M straight with seven complete branches. Hind wings with ScP and RA expanded at apex; M straight and simple without branches; CuA with five complete branches (Fig. 9F, G). ***Legs***: anteroventral margin of front femur type B_2_ (Fig. 9H). Pulvilli present on four proximal tarsomeres. Tarsal claws symmetrical and specialized, inner margin serrated, arolia present (Fig. 9I). ***Abdomen and genitalia***: eighth abdominal tergum specialized with a tuft (Fig. 9J). Supra-anal plate transverse, posterior margin convex. Paraprocts simple, splitting into two pieces, apex with tufts (Fig. 9K). Subgenital plate symmetrical. Styli similar, slender, distinctly separated; interstylar region slightly convex (Fig. 9M). Left phallomere complex, irregular bone-shaped, with three long spines (Fig. 9L). Median phallomere slender rod-shaped, apex with a slightly curved spine; accessory structure arched, a long piece of bone extends from right side of accessory structure (Fig. 9M). Hook phallomere on right side, apex curved inwards with a short spine (Fig. 9N).

**Female** similar as male.

##### Etymology.

The species name “*paratransversa*” reflects its similarity to *M. transversa* sp. nov.

##### Distribution.

China (Yunnan).

#### Margattea bicruris

Taxon classificationAnimaliaBlattodeaEctobiidae

J-J He & Z-Q Wang sp. nov.

8E349E1D-143A-521E-BA0E-00E8E0C0F468

http://zoobank.org/E491FDA2-CD3A-4B6F-B717-CB75FD06F8C4

Figure 10A–L

##### Type material.

***Holotype*:** China • ♂; Wangtianshu Scenery Spot, Mengla County, Xishuangbanna Prefecture, Yunnan Province; 23-V-2016; Lu Qiu, Zhi-Wei Qiu leg; SWU-B-EC141601. ***Paratype***: China • 2 ♂♂, same data as for holotype; SWU-B-EC141602-141603.

##### Diagnosis.

This species is similar to *M. brevialata* (Caudell, 1927) in male genitalia, but it can be differentiated from the latter by the following characters: 1) median phallomere slender rod, apex forked without spines; while in the latter, one side of splitting apex with 2 long spines; 2) left phallomere without a spine; the latter with a spine; and 3) supra-anal plate symmetrical, the front and the posterior margin straight; while in the latter, posterior margin convex, the middle part concave.

##### Measurements

**(mm).** Male (*n* = 3), pronotum: length × width 2.1–2.2 × 3.0–3.2, tegmina length: 9.9–10.9, overall length: 11.6–12.0.

##### Description.

**Figure 10.**
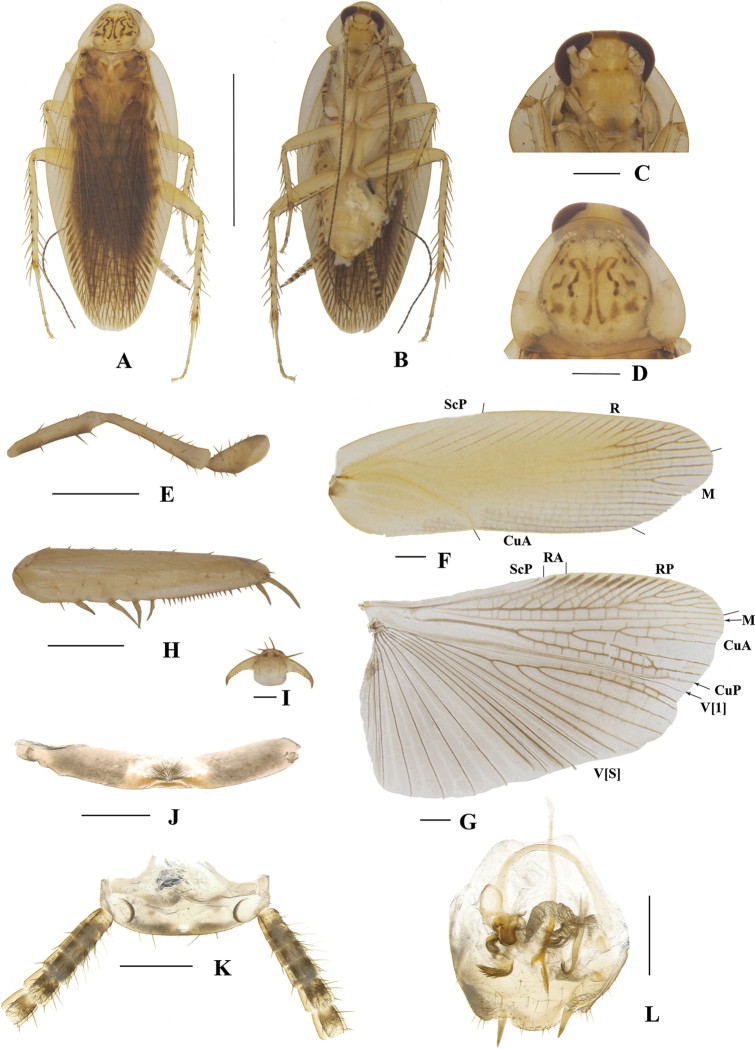
**A–L***Margattea bicruris* sp. nov., male **A** holotype, dorsal view **B** holotype, ventral view **C** head, ventral view **D** pronotum, dorsal view **E** maxillary palpi segments 3–5, ventral view **F** tegmen, dorsal view **G** hind wing, dorsal view **H** front femur, ventral view **I** tarsal claws **J** eighth abdominal terga **K** supra-anal plate and paraprocts, ventral view **L** subgenital plate and phallomeres, dorsal view. Scale bars: 5 mm (**A, B**); 0.5 mm (**C–H, J, K–L**); 0.1 mm (**I**).

**Male. *Coloration***: body pale yellow with yellowish-brown (Fig. 10A, B). Face pale yellowish-brown. Interocular space with a brown band. Ocelli spots white. Antennae pale yellowish-brown. Clypeus medium yellowish-brown (Fig. 10C). Maxillary palps light linen-colored (Fig. 10E). Pronotal disc pale yellowish-brown with yellowish-brown stripes, and two lateral light linen-colored borders (Fig. 10D). Tegmina light yellowish-brown, wings brownish grey (Fig. 10F, G). Abdomen cream-colored. Cerci yellowish-brown to pale brown (Fig. 10K). Styli faint yellow (Fig. 10L). ***Head***: vertex slightly exposed, distance between interocular shorter than antennal sockets space (Fig. 10C). Pronotum nearly trapezoidal, broader than long, the widest part after midpoint, front and posterior margins nearly straight, and postero-lateral angle blunt and round; disc with symmetrical irregular stripes (Fig. 10D). Third and fourth palpi of approximately same length, both obviously longer than fifth palp, fifth palp obviously expanded (Fig. 10E). ***Tegmina and wings***: tegmina and wings fully developed, both extending beyond the end of abdomen (Fig. 10A, B). Tegmina with Scp simple, R multi-branched, M straight with five complete branches. Hind wings with ScP and RA expanded at apex; M straight and simple, without branches; CuA with six complete branches (Fig. 10F, G). ***Legs***: anteroventral margin of front femur type B_2_ (Fig. 10H). Pulvilli present on four proximal tarsomeres. Tarsal claws symmetrical and specialized, inner margin serrated, arolia present (Fig. 10I). ***Abdomen and genitalia***: eighth abdominal tergum specialized with a tuft (Fig. 10J). Supra-anal plate transverse. Paraprocts simple, similar, splitting into two pieces (Fig. 10K). Subgenital plate symmetrical. Styli similar, slender, distinctly separated. Left phallomere complex, irregular bone-shaped. Median phallomere slender rod-shaped with apex forked; the accessory structure arched, at leftmost end with a brush. Hook phallomere on right side, apex curved inwards with a short spine (Fig. 10L).

**Female** unknown.

##### Etymology.

The Latin name “*bicruris*” refers to the median phallomere having the base forked.

##### Distribution.

China (Yunnan).

## Discussion

The number of *Margattea*MOTUs (21) recovered from GMYC and bPTP analysis were greater than the number of species (16) determined by morphological characters. Of these, 13 MOTUs totally correspond to 13 species, while the remaining three species were overestimated as eight MOTUs. The ABGD method yielded 15 MOTUs because two morphospecies were considered as one MOTU. After re-examining the specimens, we still adhere to the morphological hypotheses, that is, 16 species. Our results therefore show that ABGD was, for *Margattea* with the parameters used, more in agreement with the morphological species hypotheses than the other methods tested. DNA-based identification methods were also proven to be useful in *Margattea* male and female matching. There is no denying that DNA-barcoding methods have performed well in the rapid identification and assessment of species diversity, in finding cryptic species, and in the matching of males and females (Yang et al. 2019; Li et al. 2020). However, when there is a divergence between the morphology and molecular results, we need to look for morphological evidence to show which approach is best supported.

**Figure 11.**
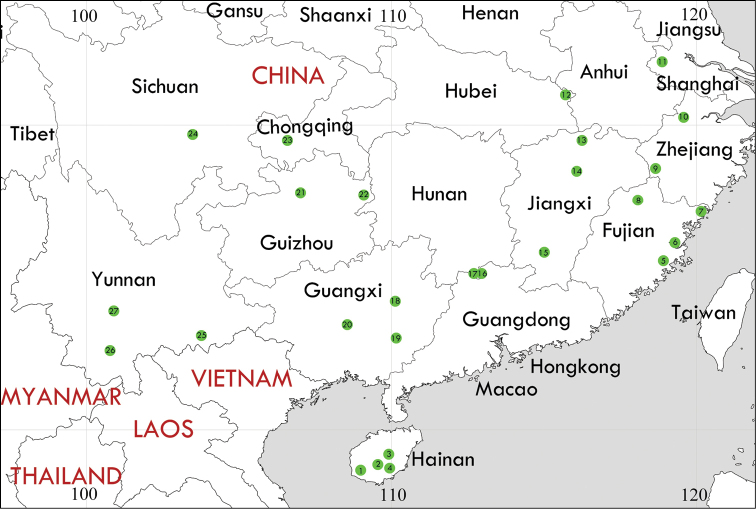
Twenty-seven collecting locations of *Margattea* species in China. The location corresponding to each number on the map was shown in Suppl. material 2: Table S2. The map originates from https://www.simplemappr.net/.

For this group of cockroaches in our study, the intraspecific and interspecific K2P genetic distances (0.0–5.9% and 4.9–25.2%, respectively) were more or less similar to values found for other cockroach groups (*Cryptocercus*: 0.00–0.61% and 2.18–20.36% (Bai et al. 2018); Ectobiidae: 0.0–7.0% and 4.6–30.8% (Che et al. 2017)). There is an overlap, also known as no barcoding gap, between the intraspecific and interspecific distance according to our results; but this barcoding gap was treated as an artifact of insufficient sampling across lycaenid butterfly taxa by Wiemers et al. (2007). The maximum intraspecific genetic distance (5.9%) existed in *M. bisignata* samples. Four MOTUs were suggested within this species in the GMYC and bPTP analyses. No obvious variation could be discerned in these different geographical populations (Fig. 11) using morphological characters, including male genitalia, in spite of this larger genetic distance (Fig. 2). Therefore, we speculate that sufficient sampling of *M. bisignata* locations resulted in greater genetic distance. While the two morphospecies with an interspecific genetic distance of 5% were hypothesized as a single MOTU in ABGD, they did have obvious and stable morphological differentiation characters, which may be the result of insufficient sampling or rapid morphological differentiation.

## Supplementary Material

XML Treatment for Margattea deltodonta

XML Treatment for Margattea cuspidata

XML Treatment for Margattea caudata

XML Treatment for Margattea disparilis

XML Treatment for Margattea transversa

XML Treatment for Margattea paratransversa

XML Treatment for Margattea bicruris

## Supplementary materials

Supplementary material 1 Table S1. Interspecific and intraspecific genetic distances Data type molecular data https://binary.pensoft.net/file/541179This dataset is made available under the Open Database License (http://opendatacommons.org/licenses/odbl/1.0/). The Open Database License (ODbL) is a license agreement intended to allow users to freely share, modify, and use this Dataset while maintaining this same freedom for others, provided that the original source and author(s) are credited.Jia-Jun He, Du-Ting Jin, Yi-Shu Wang, Yan-Li Che, Zong-Qing Wang

Supplementary material 2 Table S2. 27 collecting locations of *Margattea* species in China. Data type occurrence https://binary.pensoft.net/file/541180This dataset is made available under the Open Database License (http://opendatacommons.org/licenses/odbl/1.0/). The Open Database License (ODbL) is a license agreement intended to allow users to freely share, modify, and use this Dataset while maintaining this same freedom for others, provided that the original source and author(s) are credited.Jia-Jun He, Du-Ting Jin, Yi-Shu Wang, Yan-Li Che, Zong-Qing Wang

Supplementary material 3 Figure S1 Data type image https://binary.pensoft.net/file/541181This dataset is made available under the Open Database License (http://opendatacommons.org/licenses/odbl/1.0/). The Open Database License (ODbL) is a license agreement intended to allow users to freely share, modify, and use this Dataset while maintaining this same freedom for others, provided that the original source and author(s) are credited.Jia-Jun He, Du-Ting Jin, Yi-Shu Wang, Yan-Li Che, Zong-Qing Wang

Supplementary material 4 Raw data Data type species data https://binary.pensoft.net/file/541182This dataset is made available under the Open Database License (http://opendatacommons.org/licenses/odbl/1.0/). The Open Database License (ODbL) is a license agreement intended to allow users to freely share, modify, and use this Dataset while maintaining this same freedom for others, provided that the original source and author(s) are credited.Jia-Jun He, Du-Ting Jin, Yi-Shu Wang, Yan-Li Che, Zong-Qing Wang
