# Supplementary figures and images for: Species delimitation of Margattea cockroaches from China, with seven new species (Blattodea, Ectobiidae, Pseudophyllodromiinae)
Source: Zookeys. 2021 May 10;1036:121–51. doi: 10.3897/zookeys.1036.63232 (PMC8128847; doi:10.3897/zookeys.1036.63232)

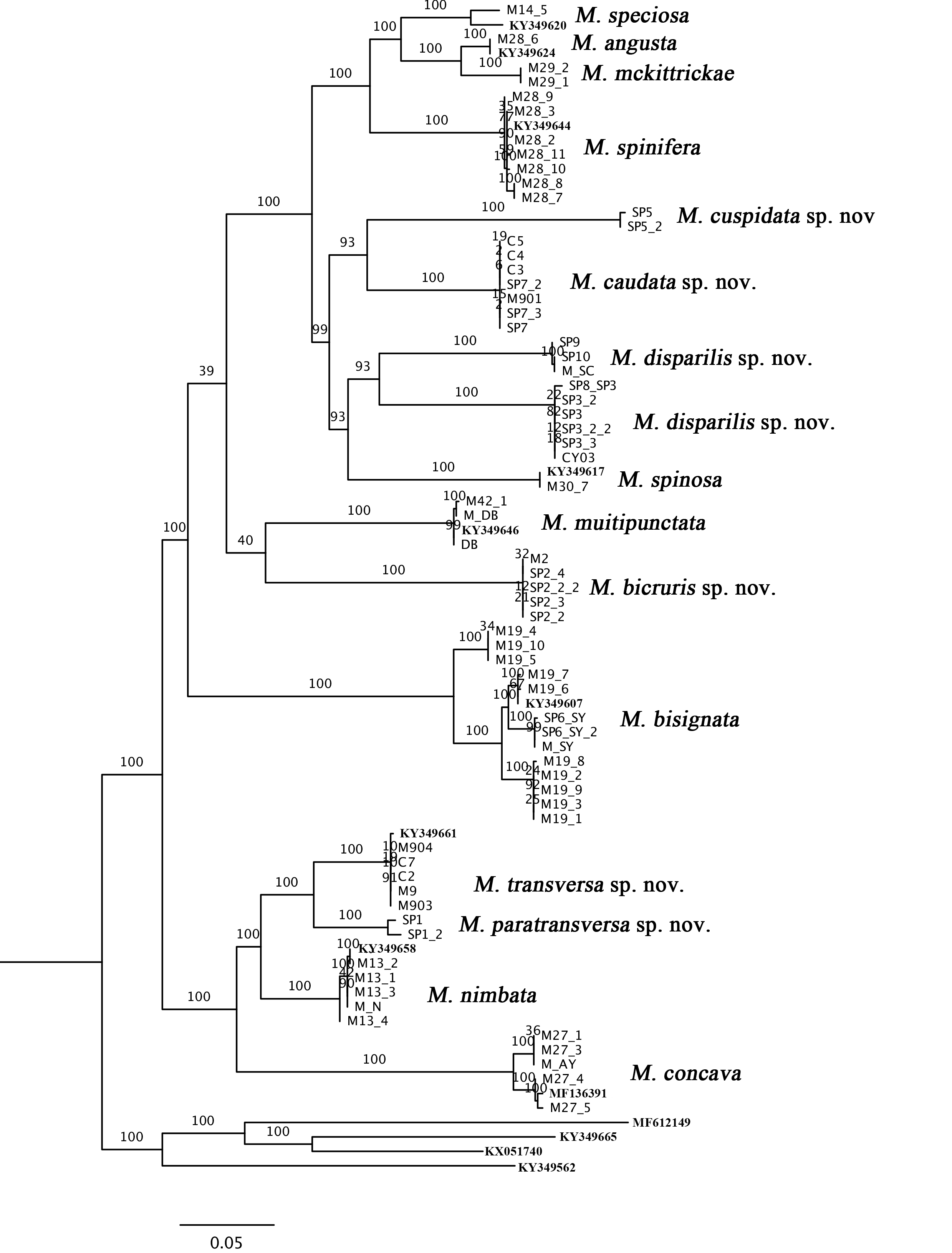

Supplement: Supplementary material 3 — Figure S1 [file zookeys-1036-121-s003.jpg]
